# Through translational prospective study, the *GSTP1* Ile105Val polymorphism emerges as prognostic marker in *de novo* large B-cell lymphoma patients

**DOI:** 10.1038/bcj.2017.38

**Published:** 2017-04-28

**Authors:** M T Delamain, E C M Miranda, G J Lourenço, C A de Souza, C S P Lima

**Affiliations:** 1 Hematology and Hemotherapy Center, University of Campinas, Sao Paulo, Brazil; 2 Department of Internal Medicine, Faculty of Medical Sciences, University of Campinas, Sao Paulo, Brazil; 3 Department of Internal Medicine, Hematology and Hemotherapy Center, Faculty of Medical Sciences, University of Campinas, Sao Paulo, Brazil

Diffuse large B-cell lymphoma (DLBCL) is a heterogeneous group of non-Hodgkin lymphoma (NHL), and its current standard of care is chemoimmunotherapy with rituximab, cyclophosphamide, doxorubicin, vincristine and prednisone (R-CHOP). Clinic-pathological markers such as the International Prognostic Index in rituximab era (R-IPI) and β2-microglobulin serum level (B2M) have been used as prognostic factors in DLBCL,^1, 2^ but do not predict patients' outcome in several cases.

Glutathione S-transferase (*GST) M1*,*T1* and *P1* detoxify chemotherapeutic agents, such as cyclophosphamide (CP), doxorubicin and/or their metabolites, by conjugating them to glutathione.^3^ GSTP1 also regulates cellular chemical stress and death through interaction with the c-Jun Nh2-terminal kinase (JNK1) protein.^4^ *GSTM1*, *GSTT1* and *GSTP1* are encoded by polymorphic genes. Homozygous deletion of *GSTM1* and *GSTT1* genes results in loss of functional enzyme activity.^5^ The *GSTP1*isoleucine (Ile) 105 valine (Val) polymorphism influences enzyme activity; protein encoded by the Ile allele is more efficient in detoxification than that produced by the Val allele.^6, 7^

Studies conducted in patients with follicular lymphoma^7^ and DLBCL^8, 9^ treated with CP-based regimens have shown that deletion of *GSTM1* and/or *GSTT1* confers worse event-free survival (EFS) and more adverse effects when compared with undeleted genes. On the other hand, breast cancer patients with the ValVal genotype of the *GSTP1* Ile105Val polymorphism had longer overall survival (OS),^10, 11^ better therapy response and reduced risk of myelosuppression^12, 13^ than those with the IleIle genotype when treated with CP-based chemotherapy.

The *GSTM1*, *GSTT1* and *GSTP1* Ile105Val polymorphisms had their roles analysed in the outcome of *de novo* DLBCL patients treated with R-CHOP in this translational prospective study.

Between July 2009 and July 2015, 144 *de novo* DLBCL patients diagnosed at the University of Campinas were included in study after approval of the Local Ethics Committee. Lymphoma diagnosis and classification were based on the 2008 World Health Organization criteria.

Patients were stratified by age, gender, serum B2M and albumin levels, Ann Arbor stage, R-IPI and National Comprehensive Cancer Network International Prognostic Index. Patients with stage I or II tumours received four cycles of R-CHOP, and six cycles of R-CHOP were administered to patients with stage III or IV tumours. Adverse events reported for each cycle of treatment were recorded according to the Common Terminology Criteria for Advance Events of the National Cancer Institute (CTCAE v.4 2009). Response to R-CHOP was scored according to the International Working Group criteria.

Genotypes were obtained in genomic DNA of patients' peripheral blood samples. Homozygous deletions of the *GSTM1* and *GSTT1* were simultaneously determined by a multiplex PCR method.^14^ The *GSTP1* Ile105Val polymorphism was analysed by PCR followed by enzymatic digestion.^15^ Positive and negative controls were used in all reactions. Replicates were performed in 10% of the reactions, achieving 100% concordance.

All analyses were conducted according to their assumptions. Logistic regression model assessed possible associations between genotypes and clinic-pathological features. Kaplan–Meier method was applied to EFS and OS, where date of diagnosis was the baseline to calculate the time, EFS until first event date (relapse, progression or death) or last seen date, whereas, OS until death or last seen date. Hence, it was applied the Cox regression for EFS and OS. To ensure the stability of model was used the bootstrapping (*n*=1000) based on repeatedly random sampling, applying the bias-corrected and accelerated method. *P*-values were two-sided, considering significantly when ⩽0.05 using the SPSS 21.0 software (IBM Corp., Armonk, NY, USA).

Table 1 presents toxicity, response rate and final status by GST genotypes distributions of 144 patients. On December 2015, 97 patients were alive (just one with disease) and 47 died (21 due to toxicity, 25 of disease progression and 1 of unrelated cause). Until here, these data were similar to those previously published.

No association of clinic-pathological features and *GSTM1* and *GSTT1* genotypes were observed. Regarding *GSTP1* Ile105Val polymorphism, the IleIle genotype was more frequent in patients who presented grade III or IV toxicity (most myelosuppression), in patients who did not obtain complete response to R-CHOP and in patients who advanced to death than in those with the remaining genotypes of the gene; carriers of the IleIle genotype were under a 2.94-, 2.18- and 2.80-fold increased risks of toxicity, not achieving complete response to chemoimmunotherapy and evolving to death (logistic regression analysis). All associations were confirmed by bootstrapping method (Table 1). Only Korean DLBCL patients with *GSTT1* null genotype and *GSTM1/GSTT1* null combined genotype displayed more frequent grade III–IV R-CHOP-related myelosuppression than those with undeleted genes, but excesses of patients with ECOG >2 and IPI 3–5 scores were identified in the *GSTT1* null genotype group. Whereas, the treatment response rate in these patients did not differ according to *GSTM1*, *GSTT1* and *GSTP1* polymorphisms.^8^

It is well plausible that patient with the *GSTP1* 105IleIle genotype has worse response and reduced toxicity when exposed to CP, since the Ile allele encodes a more efficient protein in detoxification of chemical agents than that produced by the Val allele^6, 7^ with short exposure of cells to the drug. Nevertheless, an inverse association of *GSTP1* Ile105Val polymorphism and toxicity was found in this study: the IleIle genotype was associated with higher toxicity when compared with the IleVal or ValVal genotypes. Yao *et al.*^12^ and Sugishita *et al.*^13^ described associations of the IleIle genotype with increased risk of grade III–IV myelotoxicity in breast cancer patients treated with CP. This finding cannot be explained by direct drug detoxification function of *GSTP1*; a possible alternative mechanism for this unexpected association is through the novel role of *GSTP1* in cellular stress response signalling as an inhibitor of c-Jun N terminal kinase (JNK). JNK has been implicated in proapoptotic signalling and may be required for the induced cytotoxicity of a variety of chemotherapy agents. Phosphorylation of c-Jun activates JNK resulting in subsequent activation of downstream effectors. In non-stressed cells, low JNK1 catalytic activity is orchestrated and maintained through its sequestration within the protein complex that includes *GSTP1* and JNK. However, under conditions of oxidative or chemical stress, a dissociation of the *GSTP1*:JNK complex occurs releasing *GSTP1* for oligomerisation, and the activation of released JNK allows for the subsequent induction of apoptosis.^4^ It was also speculated that the more active Ile allele results in decreased JNK activity and reduced expression of downstream cellular stress defense genes, which may predispose cells to chemical cytotoxicity.^16^

With a median follow-up of 42 months (13–83), the 5-year EFS and OS for patients were 63% and 64%, respectively. At this time, EFS and OS were shorter in patients with abnormal B2M (EFS: 55 abnormal B2M vs 88% normal B2M, *P*=0.005; OS: 58 abnormal B2M vs 88% normal B2M, *P*=0.005), albumin levels <3.5 mg/dl (EFS: 51 albumin <3.5 mg/dl vs 74% albumin >3.5 mg/dl, *P*=0.003; OS: 53 albumin <3.5 mg/dl vs 75% albumin >3.5 mg/dl, *P*=0.001), R-IPI (EFS: 46 poor, R-IPI vs 75% good/very good, R-IPI, *P*<0.0001; OS: 47 poor, R-IPI vs 76% good/very good, R-IPI, *P*<0.0001), grade III–IV toxicity (EFS: 35 grade III–IV vs 97% grade 0–II, *P*<0.0001; OS: 40% grade III–IV vs 98% grade 0–II, *P*<0.0001) and *GSTP1* IleIle genotype (EFS: 49 IleIle genotype vs 71% IleVal/ValVal genotype, *P*=0.009; OS: 51 IleIle genotype vs 72% IleVal/ValVal genotype, *P*=0.008). Differences between groups remained the same in univariate analysis; abnormal B2M and albumin, poor R-IPI, and IleIle genotype were adverse factors for EFS and OS in multivariate analysis. The IleIle genotype confirmed also having a shorter survival applying the bootstrapping method (Table 2).

Unfavourable outcome for IleIle genotype patients may be attributed to a short exposure of cells to CP^5, 6^ and lower antioxidant cellular response,^16^ with consequent disease progression and toxicity to therapy, respectively. *GSTM1* and *GSTT1* double null genotype was associated with shorter EFS in males with DLBCL in Korean DLCBL patients with a median follow-up of 15 months, but OS was not altered by *GSTP1* Ile105Val polymorphism.^8^

Differences in associations of *GSTM1*, *GSTP1* and *GSTP1* Ile105Val polymorphisms with clinic-pathological aspects and survival found herein and in Korean study^8^ may be attributed to distinct sample sizes and median time of follow-up, which were about 1.5 and 2.6 times higher in our study. The imbalance of patients with unfavourable prognosis in groups with *GSTT1* null genotype and with undeleted genes in Korean study and distinct frequencies of deleted *GSTM1* and *GSTT1* genes and *GSTP1* Ile105Ile genotype in Korean patients and in our patients may also constitute plausible explanations for differences found in both studies.

In summary, despite of some known limitations in this kind of studies, our data present preliminary evidence that *GSTP1* Ile105Val polymorphism influences toxicity and response to R-CHOP as well as survival, and it acts as an independent prognostic marker in *de novo* DLBCL.

## Figures and Tables

**Table 1:** Toxicity, response rate and final status by *GST* genotypes distributions in diffuse large B-cell lymphoma patients

*Variables*	*Patients*	*GSTM1*			*GSTT1*			*GSTP1 Ile*^*105*^*Val*		
	n*(%, range)*	*Present*	*Null*	P	*Present*	*Null*	P	*IleIle*	*IleVal/ValVal*	P
Total	144 (100.0)	83 (57.6)	61 (42.4)		123 (85.4)	21 (14.6)		53 (36.8)	91 (63.2)	
										
*Toxicity*^a^										
Grade 0–II	72 (66.0)	46 (55.4)	26 (42.6)		63 (51.2)	09 (42.8)		19 (26.4)	53 (73.6)	0.01^a^
Grades III or IV	37 (34.0)	18 (21.6)	19 (31.1)	0.15	31 (25.2)	06 (28.5)	0.57	19 (51.4)	18 (48.6)	
										
*Complete response*										
Yes	102 (71.0)	60 (72.2)	42 (68.8)	0.71	88 (71.5)	14 (66.6)	0.61	32 (31.4)	70 (68.6)	0.03^b^
No	42 (29.0)	23 (27.7)	19 (31.1)		35 (28.4)	07 (33.3)		21 (50.0)	21 (50.0)	
										
*Final status*										
Alive	97 (67.0)	57 (68.6)	40 (65.5)	0.45	82 (66.6)	15 (71.4)	0.82	28 (28.9)	69 (71.1)	0.006^c^
Dead	47 (33.0)	26 (31.3)	21 (34.4)		41 (33.3)	06 (28.5)		25 (53.2)	22 (46.8)	

Abbreviation: CI, confidence interval.

Toxicity was evaluated based on the National Cancer Institute Criteria;^16^ grade III or IV myelotoxicity was seen in 35 cases, cardiotoxicity in 1 case and nephrotoxicity in 1 case.

Response to therapy was scored according to the International Working Group Criteria; partial response, refractory and no evaluable were seen in 3, 15 and 24 cases, respectively.

Carriers of the *GSTP1* IleIle genotype were under a 2.94- (95% CI: 1.28–6.76), a 2.18- (95% CI: 1.05–4.56) and a 2.80 (95% CI: 1.36–5.76)-fold increased risks of myelotoxicity, not achieving complete response to chemoimmunotherapy and to evolve to death, respectively. (^a^)*P*_bootstrap_=0.01; (^b^)*P*_bootstrap_=0.03; (^c^)*P*_bootstrap_=0.008.

a There are some missing values.

**Table 2:** Final model for event-free survival and overall survival using Cox regression

*Features*	*Multivariate Cox analysis*				
	*Patients*	*EFS*	P	*OS*	P
	n*(%)*	*HR (95% CI)*		*HR (95% CI)*	
*B2M*					
Normal	24 (19.6)	Reference	0.002	Reference	0.002
Abnormal	98 (80.3)	1.98 (1.27–3.06)		1.99 (1.29–3.08)	
					
*Albumin*					
<3.5 g/dl	67 (51.9)	2.08 (1.09–3.98)	0.025	2.16 (1.11–4.21)	0.023
⩾3.5 g/dl	62 (48.0)	Reference		Reference	
					
*R-IPI*					
Very good/good	75 (58.1)	Reference	0.002^a^	Reference	0.002^b^
Poor	54 (41.8)	2.61 (1.40–4.88)		2.72 (1.44–5.13)	
					
*GSTP1*					
IleIle	48 (37.2)	2.04 (1.14–3.65)	0.016^c^	2.02 (1.12–3.66)	0.020^d^
IleVal or ValVal	81 (62.7)	Reference		Reference	

Abbreviations: B2M, beta2 microglobulin; CI, confidence interval; EFS, event-free survival; HR, hazard risk; OS, overall survival; R-IPI, International Prognostic Index in rituximab era. (^a^) *P*_bootstrap_=0.002; (^b^) *P*_bootstrap_=0.007; (^c^) *P*_bootstrap_=0.008; (^d^) *P*_bootstrap_=0.018.

## References

[bib1] Sehn LH, Berry B, Chhanabhai M, Fitzgerald C, Gill K, Hoskins P et al. The revised International Prognostic Index (R-IPI) is a better predictor of outcome than the standard IPI for patients with diffuse large B-cell lymphoma treated with R-CHOP. Blood 2007; 109: 1857–1861. 17105812 10.1182/blood-2006-08-038257

[bib2] Wu L, Wang T, Gui W, Lin H, Xie K, Wang H et al. Prognostic significance of serum beta-2 microglobulin in patients with non-Hodgkin lymphoma. Oncology 2014; 87: 40–47. 24969158 10.1159/000362670

[bib3] Hayes JD, Pulford DJ. The glutathione S-transferase supergene family: regulation of GST and the contribution of the isoenzymes to cancer chemoprotection and drug resistance. Crit Rev Biochem Mol Biol 1995; 30: 445–600. 8770536 10.3109/10409239509083491

[bib4] Adler V, Yin Z, Fuchs SY, Benezra M, Rosario L, Tew KD et al. Regulation of JNK signaling by GSTp. EMBO J 1999; 18: 1321–1334. 10064598 10.1093/emboj/18.5.1321 PMC1171222

[bib5] Zimniak P, Nanduri B, Pikula S, Bandorowicz-Pikula J, Singhal SS, Srivastava SK et al. Naturally occurring human glutathione S-transferase GSTP1-1 isoforms with isoleucine and valine in position 104 differ in enzymic properties. Eur J Biochem 1994; 224: 893–899. 7925413 10.1111/j.1432-1033.1994.00893.x

[bib6] Pinto N, Luderman SM, Dolan ME. Pharmacogenetic studies related to cyclophosphamide-based therapy. Pharmacogenomics 2009; 10: 1897–1903. 19958089 10.2217/pgs.09.134 PMC2820268

[bib7] Hohaus S, Mansueto G, Massini G, D'Alo F, Giachelia M, Martini M et al. Gluthathione-S-transferase genotype influence prognosis in follicular non-Hodgkin's Lymphoma. Leuk Lymphoma 2007; 48: 564–569. 17454600 10.1080/10428190601158647

[bib8] Cho HJ, Eom HS, Kim HJ, Kim IS, Lee GW, Kong SY. Glutathione S-transferase genotypes influence the risk of chemotherapy-related toxicities and prognosis in Korea patients with diffuse large B-cell lymphoma. Cancer Genet Cytogenet 2010; 198: 40–46. 20303013 10.1016/j.cancergencyto.2009.12.004

[bib9] Yri O, Ekstrøm PO, Hilden V, Gaudernack G, Liestøl K, Smeland EB et al. Influence of polymorphisms in genes encoding immunoregulatory proteins and metabolizing enzymes on susceptibility and outcome in patients with diffuse large B-cell lymphoma treated with rituximab. Leuk Lymphoma 2013; 54: 2205–2214. 23391141 10.3109/10428194.2013.774392

[bib10] Sweeney C, McClure GY, Fares MY, Stone A, Coles BF, Thompson PA et al. Association between survival after treatment for breast cancer and glutathione S-transferase P1Ile105Val polymorphism. Cancer Res 2000; 60: 5621–5624. 11059750

[bib11] Yuan P, Yuan L, Xu BL, Wang CZ, Yang HZ, Li Y. Predictive potential role of glutathione S-transferases polymorphisms in response to chemotherapy and breast cancer prognosis. Genet Mol Res 2015; 14: 16675–16681. 26681014 10.4238/2015.December.11.15

[bib12] Yao S, Barlow WE, Albain KS, Choi JY, Zhao H, Livingston RB et al. Gene polymorphisms in cyclophosphamide metabolism pathway, treatment-related toxicity and desease-free survival in SWOG 8897 clinical trial for breast cancer. Clin Cancer Res 2010; 16: 6169–6176. 21169260 10.1158/1078-0432.CCR-10-0281 PMC3058716

[bib13] Sugishita M, Imai T, Kikumori T, Mitsuma A, Shimokata T, Shibata T et al. Pharmacogenetic association between GSTP1 genetic polymorphism and febrile neutropenia in Japanese patients with early breast cancer. Breast Cancer 2016; 23: 195–201. 25008867 10.1007/s12282-014-0547-x

[bib14] Pemble S, Schroeder KR, Spencer SR, Meyer DJ, Hallier E, Bolt HM et al. Human glutathione S-transferase theta (GSTT1): cDNA cloning and the characterization of a genetic polymorphism. Biochem J 1994; 300(Pt 1): 271–276. 8198545 10.1042/bj3000271 PMC1138152

[bib15] Ribraq V, Koscielny S, Casasnovas O, Cazeneuve C, Brice P, Morschhauser F et al. Pharmacogenetic study in Hodgkin lymphomas reveals the impact of UGT1A1 polymorphisms on patient prognosis. Blood 2009; 113: 3307–3313. 18768784 10.1182/blood-2008-03-148874

[bib16] Ishimoto TM, Ali-Osman F. Allelic variants of the human glutathione S-transferase P1 gene confer differential cytoprotection against anticancer agents in Escherichia coli. Pharmacogenetics 2002; 12: 543–553. 12360105 10.1097/00008571-200210000-00006

